# Using fNIRS to study Mother-Child Brain-to-Brain Synchrony in Typical and Atypical Contexts

**DOI:** 10.1192/j.eurpsy.2022.180

**Published:** 2022-09-01

**Authors:** G. Esposito

**Affiliations:** University of Trento, Psychology And Cognitive Sciences, Rovereto, Italy

**Keywords:** Mother-child, fNIRS, Hyperscanning

## Abstract

A potential avenue of investigating the caregiver-infant relationship lies in caregiver-infant synchrony, which refers to the coordinated interplay of behavioural and physiological signals reflecting the bi-directional attunement of one individual to the other’s psychophysiological, cognitive, emotional and behavioural state. Here, we aim to study how early naturalistic caregiver-infant interactions give raise to caregiver-infant attachment, which influences physiological and psychological processes by modulating brain sensitivity. Furthermore, we aim to study how caregiver-infant bond shapes neural pathways involved in socio-emotional regulation in typical and atypical contexts. We present new evidence from fNIRS hyperscanning studies, where we measured simultaneous caregiver (mothers, N=30 and fathers N=38) and child brain activity (N=70). From the mother-child hyperscanning study (Azhari et al., 2019; 2020; 2021), we have found that higher levels of parenting stress are correlated to lower mother-child brain-to-brain synchrony, especially in the areas of the medial left prefrontal cortex. Additionally, maternal anxious attachment (Azhari et al., 2020a) also correlated in lower mother-child synchrony in the frontal and medial left prefrontal regions. These areas contain structures implicated in the inference of mental states and social cognition, highlighting the role of psychological factors such as parenting stress and attachment style in the influence of caregiver-infant bond formation during naturalistic interactions. From the combined mother- and father-child free play sessions (Azhari et al., 2020b), behavioural data revealed that parenting stress and caregivers’ recall of their past bonding experiences their own parents interact with each other to influence the eventual quality of dyadic interaction with their child.

**Disclosure:**

No significant relationships.

